# Factors Influencing the Information Support Provided by Health Care Professionals to Patients in a Dialysis Center Regarding Kidney Transplantation: A Nationwide Study

**DOI:** 10.3389/ti.2025.14159

**Published:** 2025-05-14

**Authors:** Paulina Kurleto, Lucyna Tomaszek, Irena Milaniak, Grażyna Dębska, Edyta Turkanik, Barbara Siekierska, Roman Danielewicz, Alicja Dębska-Ślizień

**Affiliations:** ^1^ Faculty of Health Sciences, Andrzej Frycz Modrzewski Krakow University, Krakow, Poland; ^2^ Department of Surgical and Transplant Nursing, Medical University of Warsaw, Warsaw, Poland; ^3^ Department of Nephrology, Transplantology and Internal Diseases, Faculty of Medicine, Medical University of Gdansk, Gdansk, Poland

**Keywords:** kidney transplant, education and training, healthcare professionals, dialysis, information support

## Abstract

For patients undergoing renal replacement therapy, kidney transplantation (KTx) is the preferred therapeutic method. The aim of this study was to investigate selected factors affecting the information support provided by healthcare professional to patients in dialysis center regarding KTx. A multiple logistic regression was carried out to assess the relationship between information support, socio-demographic factors, life satisfaction (Satisfaction with Life Scale), self-esteem (Self-Esteem Scale), perceived self-efficacy (General Self-Efficacy), attitude, knowledge about organ transplantation. Of the 1,093 respondents aged 22–72 years, 501 respondents (45.8%) always informed patients about the possibility of treatment with KTx. Physicians vs. nurses (OR = 1.79; Cl 95%: 1.48–2.16), and those who supported legalization of unspecified living kidney donation in Poland (OR = 1.30; Cl 95%: 1.07–1.59) and believed that blood donation is safe (OR = 1.29; Cl 95%: 1.12–1.47) were more likely to provide informational support. Knowledge level (OR = 1.32; Cl 95%: 1.18–1.47) and self-esteem (OR = 1.06; Cl95%: 1.03–1.10) correlated positively with information support. Male participants were less likely to provide informational support than females (OR = 0.78; Cl 95%: 0.62–0.99). The results reveal inadequate information provided by healthcare professional to patients about KTx. This highlights the urgent need for comprehensive educational programs.

## Graphical Abstract

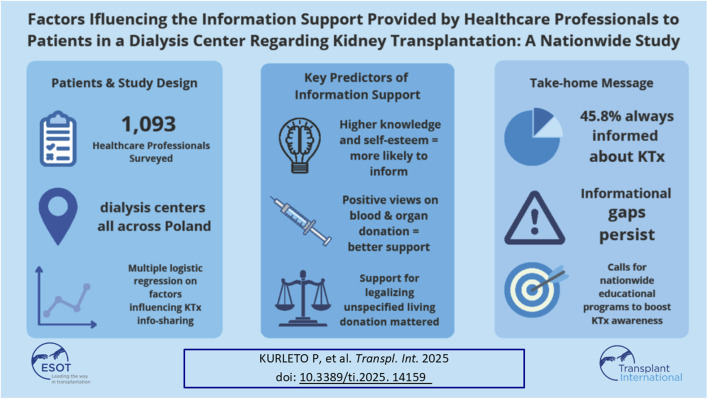

## Introduction

For 20,536 patients undergoing renal replacement therapy in the form of dialysis in Poland, kidney transplantation is the preferred therapeutic method offering improved survival and quality of life [1–3]. With the constant increase in the number of patients suffering from chronic kidney disease (CKD) and waiting for a kidney transplant, living donation has become the most important alternative in many countries [4–6]. In Poland, in 2023, kidney transplantation was performed in 3.4% of hemodialysis (HD) patients and in 13.4% of peritoneal dialysis patients (PD) [1], of which 963 (24.27 pmp) organs came from deceased and 78 (1.9 pmp) from living donors (7% of all kidney transplants, which is a record rate since the beginning of the living donation program) [7]. For comparison, other European countries, such as the Netherlands or the United Kingdom, had a living donor donation rate of 49.5% and 28% respectively [8]. Data on the number of dialysis patients and people registered on the national transplant waiting list show, that about 6% of patients undergoing renal replacement therapy are on the transplant waiting list [7].

Given these data, every effort should be made to continue to increase the rate of kidney transplantation, with particular emphasis on living donors. However, each patient and potential donor must weigh the benefits of transplantation against the potential risks of the procedure in order to choose a treatment method. Scientific studies have shown, that patient education is an important component of informed decision-making regarding the treatment of ESRD (9); however, there is evidence that patients do not have adequate knowledge about kidney transplantation [9–11]. Therefore, the tasks of medical personnel in the era when kidney transplantation is considered the best therapeutic method include, among others, presenting possible therapeutic options, including the option of kidney transplantation. In practice, nephrologists are often the first to inform patients about the possibility of treatment with a kidney transplant from a living or deceased donor. Based on the circumstances, the qualification process may begin in a nephrology department or in a dialysis center. A conversation with the patient and family about a potential living donation is obligatory in Poland. The reporting physician must note this fact when entering the patient onto the National Waiting List. After qualifying for a kidney transplant from a deceased or living donor, the potential kidney recipient is placed on the National Waiting List [12].

According to a study by Kucirka et al. from 2012, as many as 30.1% of patients were not informed by nephrologists about the possibility of kidney transplantation in the initial phase of end-stage renal failure [13]. Additionally, studies conducted by Waterman et al. in 2012 show that dialysis center staff were only able to correctly answer questions regarding knowledge about kidney transplantation in 50% of cases, and some employees admitted that they still have very limited time to educate dialysis patients and their families. Moreover, almost one third (30%) declare, that they do not have sufficient knowledge to conduct such education [14]. There are reports in the scientific literature identifying many barriers that hinder effective education about transplantation, including time constraints and poor access to educational materials, as well as barriers that make it difficult for patients to learn, such as fear or lack of trust in medical personnel [9].

The Acts on the Medical and Nursing Professions define their competencies in providing patient care. The doctor explains the patient’s health condition and discusses the therapeutic process in detail. The nurse provides information about the patient’s health condition to the extent necessary to provide nursing care, health education, and health promotion [15, 16]. According to the educational model proposed by the University Clinical Centre in Gdańsk, educational training is offered to all patients with CKD at any stage of the disease (and their families), considering renal replacement therapy, including kidney transplantation. An interdisciplinary educational team comprises of four nurses, four doctors, and a dietician [17]. The primary members of the educational team are a nurse and a nephrologist. However, the nurse is usually the patient’s primary contact person and is coordinating patient care [18]. In Poland, organized education on renal replacement therapy is conducted mainly by dialysis centers or clinical nephrology centers with dialysis therapy [17, 19].

Due to the increasing number of dialysis patients and the possibility of becoming a potential kidney recipient those awaiting kidney transplantation a study was designed and conducted to show the attitudes and knowledge on kidney transplantation among dialysis center staff and to identify educational methods used in dialysis centers. A more comprehensive understanding of the attitudes of medical personnel towards transplantation, including living donation, will allow for the preparation of public health and educational programs to support living kidney donation. It is worth emphasizing the scientific value of the study, as it is the first one conducted on such scale, gathering data from all Polish voivodeships and in regards of the number of studied personnel.

## Material and Methods

### Study Desing, Setting

A cross-sectional study was conducted between February 2023 and June 2024 after obtaining the consent of the Bioethics Committee of the Andrzej Frycz Modrzewski Krakow University (decision no. KBKA/3/O/2023). The study included a group of 1,093 employees (physicians and nurses) from public and private dialysis centers across Poland. The guidelines of the Helsinki Declaration (World Medical Association, 2013) and STROBE (Strengthening the Reporting of Observational Studies in Epidemiology) [20], as well as The General Data Protection Regulation [21] were followed. The study was registered at ClinicalTrials.gov (ID: NCT05797337).

### Participants

We conducted a cross-sectional study among specialists working in dialysis centers throughout Poland. The study involved employees who voluntarily agreed to participate in the study, had a communicative knowledge of Polish, and had the right to practice medicine or nursing. We assumed that education is delivered in a dialysis centre by a multidisciplinary team including, a nephrologist, and a nurse [17–19]. Before starting the study, each participant received comprehensive information about the purpose and course of the study.

### Instruments

The study used a diagnostic survey with a questionnaire technique. The questionnaires were distributed in paper and online form. The researchers sent 1,451 paper surveys to 74 dialysis centers across the country, where consent was obtained from the facility director and the head of the dialysis center. The online surveys were obtained in cooperation with the industry publishing house Practical Medicine (Medycyna Praktyczna). Additionally, dialysis center employees were encouraged to use the snowball method. The study used a self-assessment questionnaire, which included, among others, a socio-demographic data sheet and questions regarding the respondents’ attitudes towards kidney transplantation, knowledge in this area and educational methods used in the facilities where they provide care for dialysis patients. Overall knowledge scores being a sum of correct answers (“definitely yes”) to 5 questions from a given area (questions 27–31) ranged from 0 to 5 pts. The higher the score, the better the knowledge. For knowledge evaluation, the following statements were presented: 1) Kidney transplantation contributes to the quality of life of patients with chronic kidney disease; 2) Kidney transplantation is a better therapeutic method than dialysis therapy; 3) Kidney transplantation from a living donor is more beneficial for recipients than transplantation from a deceased donor; 4) Kidney transplantation from a living donor can pose a major threat for the donor’s health and life; 5) Kidney transplantation from a living donor will significantly deteriorate the donor’s quality of life.

Standardized tools were the Polish version [22, 23] of the Satisfaction with Life Scale [24]. The Satisfaction with Life Scale contained five statements in which the respondent assessed the extent to which each of them referred to his or her life so far. The responses were measured on a 7-point Likert scale: 7 – strongly agree, 6 – agree, 5 – somewhat agree, 4 – neither agree nor disagree, 3 – somewhat disagree, 2 – disagree, 1 – I definitely disagree. The measurement result was a general indicator of the sense of satisfaction with life ranging from 5 to 35 points (a score of 20 is considered neutral). The instrument is characterized by good psychometric properties. Internal consistency measured by Cronbach’s alpha was 0.86. The test-retest stability of the results was satisfactory (0.85–0.93 in three-week intervals, 0.87–0.88 in six-week intervals and 0.86 in nine-week intervals). The higher the score, the higher the life satisfaction.

Self-esteem was measured using the Rosenberg Self-Esteem Scale (RSES) [25] RSES is a 10-item scale that measures global self-worth by evaluating positive and negative feelings about one’s self. The responses were measured on a 4-item Likert scale: 0 (strongly agree), 1 (agree), 2 (disagree), and 3 (strongly disagree). Five of the items are positively worded (items 1, 2, 4, 6, and 7) whereas the remaining five are negatively worded (3, 5, 8, 9, and 10). The maximum score is 30, where higher scores indicate higher self-esteem. The range of possible results is from 0 to 30 points. Raw results were converted into standard units on the sten scale. The SES scale has good psychometric properties, with Cronbach’s alpha ranging from 0.81 to 0.83. The Rosenberg self-assessment scale was used in the Polish adaptation of Dzwonkowska et al. [26].

The Generalized Self-Efficacy Scale (GSES) by Schwarzer and Jerusalem (Polish adaptation: Juczyński) was also used as a standardized tool to measure generalized self-efficacy; the scale consists of 10 statements that form one factor, and the results are calculated according to a key that should be interpreted in relation to sten norms; the Polish version of the scale has good psychometric properties, Cronbach’s alpha coefficient = 0.85 [24].

### Statistical Analysis

Qualitative variables were presented as the frequency of a given category and its corresponding percentage, while quantitative variables were showed as medians (upper and lower quartiles) and means (standard deviations). Intergroup differences for qualitative data were assessed using the Chi-square test, while for quantitative variables using Mann–Whitney test. Spearman correlation analysis was used to assess the relationship between two quantitative variables. The correlation coefficient (R) was interpreted as: negligible (<0.1), weak (0.1–0.39), moderate (0.4–0.69), strong 0.7–0.89 and very strong (0.9–1.0). The Shapiro–Wilk test was used to examine the normality of the distribution of variables. A multiple logistic regression was carried out to assess the relationship between dependent variable “information support” (always/ not always) and independent variables such as: gender; profession; place of residence; blood donation is safe; bone-marrow transplant is safe; support for legalization of unspecified living kidney donation in Poland; consent to donate organs after death; consent to donate organs after death of a family member; acceptance for family member’s decision to donate after death; knowledge level; life satisfaction; self-esteem; perceived self-efficacy.

First, a simple logistic analysis was performed to select predictors–a variable which had a p-value <0.1 was then entered into the multiple regression model. To ensure the model’s effectiveness, backward elimination technique was utilized. The Hosmer-Lemeshow test suggested, that the model is a good fit to the data as p > 0.05. Nagelkerke’s R^2^ describes the proportion of variance in the outcome that the model successfully explains. To test the significance of individual coefficients in the model, the Wald statistics were used. The odds ratio with 95% confidence interval was also calculated. The variance inflation factor (VIF) was used to detect multicollinearity in all final regression models (VIF <5 was assumed as acceptable) [27].

Internal consistency rate of the Satisfaction with Life Scale (Cronbach’s alpha: 0.86), the Rosenberg Self-Esteem Scale (Cronbach’s alpha: 0.83), and the Generalized Self-Efficacy Scale (Cronbach’s alpha: 0.86) was estimated; a scale is considered reliable if its Cronbach’s alpha is equal to 7 or higher [23, 24]. Statistical analysis was carried out with Statistica 13.3 (^®^1984–2017 TIBCO Software Inc, Stat Soft Poland, Krakow) and Set Plus (Stat Soft Polska Sp. z o. o. 2024, Set Plus version 5.1.0.^1^). The threshold of statistical significance for all tests was set at p = 0.05.

### Outcomes

The primary outcomes described the percentage of health care professionals who always informed patients about the possibility of treatment with a kidney transplant (information formulated on the basis of the statement: information regarding kidney transplantation is provided at least once to all patients eligible for transplantation, regardless of whether they have expressed an interest in transplantation or not). The secondary outcomes included: socio-demographic factors, life satisfaction, self-esteem, perceived self-efficacy and attitude and knowledge about organ transplantation.

## Results

**FIGURE 1:**
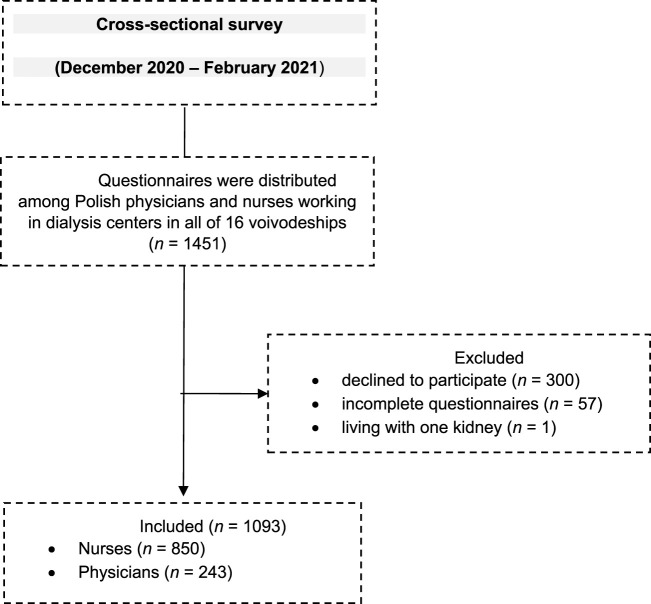
Flow diagram–participants.

Of the 1,451 nurses and physicians who were approached to participate in the study, 1,093 responses were received that met all inclusion criteria. The overall response rate was 68%. Respondents were divided into two groups: those who always do (45.8%) and those who do not always provide information support (54.2%); Figure 1.

### Participant Characteristics

The analysis included survey data of 1,093 health care professionals working in dialysis centers. The number of the nurses and physicians were 850 (77.8%) and 243 (22.2%), respectively. The female-to-male ratio was 963:130. Median age of respondents in the whole sample was 50 [43; 55] and ranged from 22 to 72 years. The vast majority of them were married or in a committed relationship (75.1%; n = 821), had children (78.4%; n = 857) and siblings (87.2%; n = 953). They were mostly urban residents (74.6%; n = 815). Median job seniority was 20 [7; 27] and the dialysis center was the main workplace for 71.4% of respondents (n = 780). Fifty-two percent (n = 568) of health care professionals worked no more than 160 h per month. Table 1 presents the socio-demographic characteristics of health care professionals who always informed patients about the possibility of treatment with a kidney transplant and those who did not. The study groups differed significantly in terms of profession, academic degrees and titles, gender, qualification training program, monthly working time, and place of residence.

**TABLE 1:** Sociodemographic characteristics of healthcare professionals who always informed patients about the possibility of treatment with a kidney transplant and those who did not.

Variables	Information support			
	Always n = 501	Not always n = 592	Statistics values	*P*-values
Age (years)				
• Me, Q_25_; Q_75_	50 [43; 55]	50 [41; 55]	Z = −0.84	0.40
• M ± SD	48.3 ± 10.5	47.6 ± 10.7		
Job seniority (years)				
• Me, Q_25_; Q_75_	20 [7; 27]	19 [7; 27]	Z = −0.76	0.45
• M ± SD	18.1 ± 11.6	17.6 ± 11.3		
Gender				
• Female	427 (85.2)	536 (90.5)	χ2 = 7.30	0.007
• Male	74 (14.8)	56 (9.5)		
Place of residence				
• City	393 (78.4)	422 (71.3)	χ2 = 7.33	0.007
• Village	108 (21.6)	170 (28.7)		
Married or in a committed relationship	377 (75.2)	444 (75.0)	χ2 = 0.009	0.92
Having children	390 (77.8)	467 (78.9)	χ2 = 0.17	0.67
Having siblings	438 (87.4)	515 (86.9)	χ2 = 0.04	0.83
The dialysis center as the main workplace	359 (71.7)	421 (71.1)	χ2 = 0.04	0.84
Monthly working time (hours)				
• ≤160	232 (46.3)	336 (56.8)	χ2 = 11.87	0.0006
• ≥161	269 (53.7)	256 (43.2)		
Profession				
• Nurses	332 (66.3)	518 (87.5)	χ2 = 70.75	<0.0001
• Physicians	169 (33.7)	74 (12.5)		
The specialization program completed	270 (53.9)	226 (38.2)	χ2 = 27.04	<0.0001
Academic degrees and titles				
• Doctor (degree)	48 (9.6)	15 (2.5)	χ2 = 30.44	<0.0001
• Doctor habilitated (degree)	6 (0.5)	3 (1.2)		
• Professor (title)	8 (1.6)	3 (0.5)		
Nurses				
Education (n = 850; 100%)				
• Master of Science in Nursing	115 (34.6)	160 (30.9)	χ2 = 2.67	0.26
• Bachelor in Nursing	111 (33.4)	165 (31.8)		
• Registered Nurse	106 (31.9)	193 (37.3)		
Specialization program (*n* = 320; 100%):				
• Nephrology nursing	63 (44.1)	78 (44.1)	χ2 = 0.00	0.99
• Internal nursing	80 (55.9)	99 (55.9)		
Qualification training program (*n* = 490; 100%)				
• Internal nursing	8 (4.0)	14 (4.8)	χ2 = 7.51	0.02
• Nephrology nursing with dialysis	186 (93.5)	277 (95.2)		
• Transplant nursing	5 (2.5)	0 (0.0)		
Physicians				
Specialization program (*n* = 176; 100%)				
• Nephrology	111 (87.4)	41 (83.7)	χ2 = 2.31	0.31
• Clinical transplantology	1 (0.8)	2 (4.1)		
• Nephrology and clinical transplantology	15 (11.8)	6 (12.2)		

Age and job seniority were presented as median [upper and lower quartile] and mean (± standard deviation). Categorical variables were presented as absolute numbers and percentages.

### Life Satisfaction, Self-Esteem, and Perceived Self-Efficacy

The median total scores of The Satisfaction with Life Scale (24 [20; 27] vs. 23 [20; 26]; Z = −3.88; p = 0.0001), Self-Esteem Scale (21 [18; 25] vs. 20 [18; 22]; Z = −5.78; p < 0.0001), and Perceived self-efficacy (30 [28; 33] vs. 29 [27; 31]; Z = −3.51; p = 0.004) among health care professionals was significantly higher in the group of health care professionals who always informed patients about the possibility of treatment with a kidney transplant than those who did not.

### Attitude Towards Organ, Blood and Bone Marrow Donation

If necessary, 85% of respondents would donate a kidney to their child, 53% to a parent, 48% to a sibling and spouse, and 6% to a stranger. The respondents most often declared that they would accept a kidney from a dead donor (50%). In case of a living transplant, 40% would accept a kidney from a spouse, 32% from a sibling, 31% from a parent, 27% from a stranger, 12% from a child, 17% were not sure whether they would accept a kidney from anyone, and 4.5% would not agree to a transplant. This question was a multiple-choice question. Almost a quarter of the whole sample (24.4%; n = 267) declared themselves a blood donor at least once in their life and 19% (n = 208) of persons registered with the bone marrow donor bank. Health care professionals who always informed patients about the possibility of treatment with a kidney transplant, were more likely to believe blood donation (69.6% vs. 48.3%; p < 0.0001) and bone-marrow transplant (56.7% vs. 36.7%; p < 0.0001) are safe than those who did not declare it. They were also determined to donate their own organs for transplantation after death (72.3% vs. 58.4%; p < 0.0001) and expressed their acceptance of organ donation from close relatives after their death (62.1% vs. 44.4%; p < 0.0001). They were also more likely to believe that - despite the principle of presumed consent - asking the family whether the deceased expressed their objection to organ donation after death during their lifetime and in the presence of two witnesses (39.5% vs. 29.2%) is necessary and should not change. They would also be more willing to support the legalization of kidney donation to a stranger in Poland (18.4% vs. 9.1%; p = 0.00001) (Table 2).

**TABLE 2:** Attitude towards organ, blood and bone marrow donation.

Variables	Information support			
	Always n = 501	not always n = 592	Statistics values	*P*-values
Blood donation is safe	348 (69.6)	286 (48.3)	χ2 = 50.45	<0.0001
Bone-marrow donation is safe	284 (56.7)	217 (36.7)	χ2 = 43.85	<0.0001
Consent to donate organs after death	362 (72.3)	346 (58.4)	χ2 = 22.68	<0.0001
Consent to donate organs after death of a family member	311 (62.1)	263 (44.4)	χ2 = 33.88	<0.0001
Accepted the family members will donate an organ	198 (39.5)	173 (29.2)	χ2 = 12.83	0.0003
Support for legalization of unspecified living kidney donation in Poland	92 (18.4)	54 (9.1)	χ2 = 20.02	0.00001
Organ trafficking risk	434 (86.6)	524 (88.5)	χ2 = 0.89	0.34

Categorical variables were presented as absolute numbers and percentages.

### Knowledge About Kidney Transplantation

Over 80% of all respondents believe that kidney transplantation definitely contributes to improving the quality of life of patients with chronic kidney disease (n = 913) and is a better therapeutic method than dialysis therapy (n = 882). Over 53% (n = 585) of respondents have doubts whether kidney transplantation from a living donor is more beneficial for the recipient than transplantation from a deceased donor. According to only 17.2% (n = 189) of respondents, kidney transplantation from a living donor definitely does not pose a significant threat and in the opinion of 23% (n = 253), kidney transplantation from a living donor will definitely not affect the deterioration of his quality of life. The knowledge of the respondents about kidney transplantation in the group that always provided information support was significantly higher than in the group that did not always give such support (median 3 [2; 4] vs. 2 [2; 3]; Z = −8.53; p < 0.0001).

Weak positive correlations were noted between variables: knowledge and job seniority (R = 0.08; t = 2.70; p = 0.01), knowledge and self-esteem (R = 0.18; t = 6.01; p < 0.0001), and knowledge and perceived self-efficacy (R = 0.14; t = 4.56; p < 0.0001).

### Transplant Education Practices

According to the vast majority of health care professionals working in dialysis centers (90.6%, n = 990), patients with end-stage renal disease are interested in kidney transplantation as a one of the treatment options. However, in the process of qualifying for kidney transplantation, the percentage of physicians and nurses declaring that they had not always talked to the recipient/family about a potential live donation was 30.4% and 60.9%, respectively.

In addition to the oral form, educational practices such as providing handouts/brochures about transplant (58.4%; n = 638), displaying transplant posters (25.3%; n = 277), providing list of transplant websites (15.3%; n = 167), organizing meetings between patients and a living kidney donor (5%; n = 55) or educational meetings about living donation for family members of patients (4.7%; n = 52) were also used. Only 16.8% (n = 184) of respondents indicated that a formal transplant education program existed in their dialysis unit.

It should be noted that most of health care professionals (71.6%; n = 783) spend very little time providing transplant education to patients (from a few minutes to half an hour). Only 39.1% (n = 95) of physicians and 9.1% (n = 77) of nurses declared sufficient knowledge of kidney transplantation and were able to answer most of the patients’ questions. It should also be noted that physicians devote more time to self-education per month (several days or more than several days) compared to nurses (31.3%, n = 76 vs. 16.9%; n = 144; χ2 = 36.06; p < 0.0001). The sources of knowledge on this subject are: scientific journals (66.3%; n = 725), textbooks (64%; n = 700), specialist/further training courses (55.5%; n = 607), personnel (47.6%, n = 520), scientific conferences (41.3%; 452), Internet portals (39.1%; n = 427), websites of scientific societies (34%; n = 374). Physicians are twice as likely as nurses to participate in scientific conferences and use websites of scientific societies.

### Factors Associated With Information Support

Table 3 shows the three multiple logistic regression models for information support. All presented models are statistically significant (p < 0.05). In all obtained regression models VIF ranged between 1.0 and 2.1, indicating that multicollinearity did not influence the regression results.

**TABLE 3:** Factors influencing the information support provided by healthcare professionals to patients in a dialysis center regarding kidney transplantation.

Variables	B	SE (B)	Wald test	p	OR (Cl 95%)
Simple logistic regression					
Male ^Reference: Female^	0.25	0.09	7.19	0.01	1.28 (1.07–1.55)
City ^Reference: Village^	0.19	0.07	7.29	0.01	1.21 (1.05–1.39)
Physician ^Reference: Nurse^	0.63	0.08	66.24	0.00	1.89 (1.62–2.20)
Blood donation is safe	0.45	0.06	49.49	0.00	1.56 (1.38–1.77)
Bone-marrow donation is safe	0.25	0.06	16.88	0.00	1.29 (1.14–1.45)
Support for legalization of unspecified living kidney donation in Poland	0.40	0.09	19.33	0.00	1.50 (1.25–1.79)
Consent to donate organs after death	0.31	0.06	22.44	0.00	1.36 (1.20–1.55)
Acceptance of organ donation following the death of a family member	0.36	0.06	33.52	0.00	1.43 (1.27–1.61)
Asking the family whether the deceased expressed their objection to organ donation after death during their lifetime and in the presence of two witnesses is necessary and should not change	0.23	0.06	12.76	0.00	1.26 (1.11–1.43)
Knowledge level	0.43	0.05	69.76	0.00	1.53 (1.39–1.70)
Life satisfaction	0.05	0.01	14.01	0.00	1.05 (1.02–1.07)
Self-esteem	0.09	0.02	30.58	0.00	1.09 (1.06–1.12)
Perceived self-efficacy	0.05	0.01	11.39	0.00	1.05 (1.02–1.08)
Multiple logistic regression model _ physicians R^2^ Nagelkerke = 0.11; Hosmer Lemeshow = 8.43; *p* = 0.39					
Knowledge level	0.42	0.11	13.97	0.00	1.53 (1.22–1.91)
Perceived self-efficacy	0.08	0.03	5.09	0.02	1.08 (1.01–1.15)
Multiple logistic regression model _ nurses R^2^ Nagelkerke = 0.11; Hosmer Lemeshow = 13.43; *p* = 0.10					
Knowledge level	0.25	0.07	15.49	0.00	1.28 (1.13–1.45)
Support for legalization of unspecified living kidney donation in Poland	0.32	0.11	8.09	0.004	1.37 (1.10–1.70)
Blood donation is safe	0.26	0.08	11.49	0.001	1.29 (1.12–1.51)
Self-esteem	0.06	0.02	9.88	0.002	1.06 (1.03–1.11)
Multiple logistic regression model_ the whole group R^2^ Nagelkerke = 0.19; Hosmer Lemeshow = 11.16; p = 0.19					
Male ^Reference: Female^	−0.24	0.12	3.98	0.046	0.78 (0.62–0.99)
Physician ^Reference: Nurse^	0.58	0.10	36.63	0.00	1.79 (1.48–2.16)
Knowledge level	0.28	0.06	25.08	0.00	1.32 (1.18–1.47)
Support for legalization of unspecified living kidney donation in Poland	0.27	0.10	7.07	0.008	1.30 (1.07–1.59)
Blood donation is safe	0.25	0.07	13.14	0.00	1.29 (1.12–1.47)
Self-esteem	0.06	0.02	13.33	0.00	1.06 (1.03–1.10)

B, Regression coefficient; SE, Standard error; OR, Odds ratio; and CI, Confidence interval.

In the case of *physicians* only knowledge level and perceived self-efficacy were statistically significant in the regression model. The contribution to the *nurse’s* model comes from knowledge level, support for legalization of kidney donation to a stranger from living donors in Poland, safety of blood donation, and self-esteem. The all parameters in above-mentioned two models are positively associated with information support.

The multiple logistic regression model developed for the *whole group* reviled that physician (vs. nurses), and those who supported legalization of unspecified living kidney donation in Poland and believed that blood donation is safe were more likely to provide informational support. Knowledge level and self-esteem correlated positively with information support. Male participants were less likely provide informational support than female.

## Discussion

The results of this study showed that physicians were more likely to provide informational support to dialysis patients than nurses. Gender differences in giving information support were recorded. Knowledge level and self-esteem correlated positively with informational support. Additionally, such support was provided by people who would support legalization of unspecified kidney donation from living donors in Poland, and believed that blood donation is safe.

The first factor “physicians were more likely to provide informational support to dialysis patients than nurses” is connected with the facts that physicians are responsible for the treatment plan and qualification and inclusion on the transplant waiting list [28, 29]. Trachtman H. et al. in their study found physicians’ support for living kidney donation as a viable medical option [30].

Oriol-Vila et al. [31], based on a review of 12 studies on the process of deceased donor transplantation showed, that after nurse educational interventions, dialysis patients and kidney transplant recipients had better health outcomes. It is therefore alarming that 30.4% of Polish physicians and 60.9% of nurses caring for patients in the dialysis center declared that they did not always inform patients about kidney transplantation as the best therapeutic option. These national data are similar to the report by Kucirka et al. [13], which showed that 30.1% of American patients with ESRD did not have information from their nephrologists in dialysis centers about the possibility of transplantation. Educational neglect is one of the main barriers to access transplantation treatment [32], because uninformed patients have limited access to the transplant waiting list and transplantation [33]. Lack of education may contribute to poorer quality of life for dialysis patients, as dialysis is not an ideal long-term solution and transplantation offers a better perspective. Furthermore, dialysis is more expensive than kidney transplantation in the long term, leading to increased treatment costs [34].

The results of the study suggest that men–both doctors and nurses–are less likely to provide informational support to patients, than women. This may be due to differences in communication style, approach to patients, social and cultural conditions. The study by Roter and Hall [35] shows that female doctors were more likely to engage in conversations with patients, show more empathy and spend more time on health education than male doctors. Street et al. [36] found that regardless of gender, doctors showed more patient-centered communication, but only with patients they perceived as better communicators, more satisfied and more likely to follow recommendations. In contrast, Younas and Sundus [37] reported that patients perceived nurses as supportive and comforting and provided them with necessary information, but many of them did not answer their questions in a timely and sufficient manner.

Transplant programs worldwide are regulated by law; however, the knowledge and attitude of professionals and general society is important to increase the number of transplants. Our study showed that knowledge level of the professionals correlated positively with informational support for the patients. On the one hand patient education requires significant resources and in addition, some studies also show that nephrologists do not consistently discuss mortality risks with patients, both in the case of dialysis patients and during the kidney transplant evaluation education process [38]. Available studies showed, that having good knowledge and good attitudes may lead to better practice in patient information about treatment options [39–41].

The another factor “support legalization of unspecified kidney donation from living donors in Poland” is important from the perspective of living donation. The rates of transplants from living donors in Poland are very low. In 2023 there were 78 kidney transplants from living donors, 5 more than in 2022 [7]. Anonymous live organ donors or unspecified donors are individuals willing to be organ donors for any transplant recipient especially kidney donor with whom they have no biological or antecedent emotional relationship [42]. Donation to a stranger is legal in numerous countries, including the USA, Canada, Australia, and Israel and European countries like: Great Britain, Sweden, or the Netherlands [43]. Unspecified living donations can help bridge transplant disparities, help mitigate the shortage of kidney grafts globally and improve organ allocation [44]. In our previous study we found that in Poland, there is a strong support for legalization of unspecified living kidney donation (60% of respondents) [45].

In our study, the positive attitude towards blood donation, especially nurses’, is the factor that affects the informational support for dialysis patients. This association can be explained by several psychological and behavioral factors. It is likely, that these individuals tend to have a greater sense of social responsibility and are more involved in promoting health literacy, including organ donation and transplantation.

Our study revealed, based on logistic regression model constructed separately for nurses and the entire group, that self-esteem correlated positively with informational support. Self-esteem is considered an important factor in human behavior and plays a significant role in the professional functioning of medical personnel, especially nurses, by influencing their interpersonal skills and the way they communicate with patients. People with low self-esteem are characterized by a lack of self-confidence, and as a result, they are unable or reluctant to communicate effectively with patients or use inappropriate communication methods [46]. People with high self-esteem believe in their own competences, which may translate into a greater willingness to provide health education in the field of kidney transplantation and thus contribute to an increase in the number of transplants and improvement in the quality of life of patients. It is worth noting that in the logistic regression model developed only for doctors, a significant factor related to informing the patient was perceived self-efficacy, defined as an individual’s belief in coping with difficult situations and obstacles [18]. A higher sense of self-efficacy increases motivation to act [47] and this probably explains the fact that doctors with a higher sense of efficacy are more likely to undertake patient education.

### Implications for Clinical Practice

In the last year, an increase in kidney transplants from a living donor has been observed in Poland. For the first time in several decades of the existence of the living donation program, a rate of 7% was achieved; previously, it was a maximum of 5% of all kidney transplants [7]. Nevertheless, this is still a low rate compared to many Western European countries [8]. The results of our study reveal inadequate information provided by healthcare professionals to patients about kidney transplantation. This highlights the urgent need for comprehensive educational programs for both healthcare professionals and patients, with a focus on the benefits of kidney transplant programs and lifetime indefinite kidney donation. To assure these programs’ effectiveness, the Polish transplant society should play the key role in developing the frameworks for such programs.

Future educational research should determine which techniques work best and how effective strategies can be made available to the entire population of patients with CKD and ESRD and their family members. Research studies confirm, that female healthcare professionals are more likely to provide informational support to patients than males. This disparity can be reduced through communication training, standardization of patient information procedures, and promotion of greater involvement of all healthcare professionals in patient education.

Research suggests that positive attitudes toward blood donation among healthcare professionals are associated with a greater likelihood of providing informational support to dialysis patients regarding kidney transplantation. Fostering a culture of blood donation awareness within healthcare teams can lead to better patient education and improved transplant outcomes.

### Strengths and Limitations

The strengths of the study is the large sample sizes and use of standardized tools. Additionally, our study is the first nationwide study on this matter. The limitation of this study is: the self-assessment questionnaire used within this study was not validated, and therefore the results must be interpreted with this in mind. In addition, the sample structure was not calculated due to the lack of detailed data on the number of nephrologists and nurses in the country working in the dialysis center. The total number of nephrologists in 2022 was 1,386 (F: 846; M: 538) and 121 (F: 99; M: 22) for pediatric nephrologists [48]. There’s however no data on if they work in dialysis centers, Nephrology Departments, or both. There is no exact information on the number of nurses working in dialysis centers, it is estimated that about 4,300 nephrology nurses work in Polish nephrology and transplant centers [49]. It is also worth noting, that many of dialysis center personnel work in more than one facility, thus it is hard to differentiate whether working in a public or private dialysis center has or has not an impact on the studied sample’s views and practices. We also are aware of the fact, that our studied group are dialysis centers only–we have not targeted the Nephrology Departments personnel–again we have not asked about working elsewhere so there is a possibility of some personnel having their answers effected by this fact.

## Conclusion

Summarizing, physicians were more likely to provide informational support to dialysis patients than nurses. Additionally, such support was provided by people who would support legalization of unspecified kidney donation from living donors in Poland, believed that blood donation is safe and would also accept their family members decision to donate an organ after death. Knowledge level and self-esteem correlated positively with informational support.

## Data Availability

The raw data supporting the conclusions of this article will be made available by the authors, without undue reservation.

## Footnotes

1
http://www.statsoft.pl
